# From Photogrammetry to Virtual Reality: A Framework for Assessing Visual Fidelity in Structural Inspections

**DOI:** 10.3390/s25144296

**Published:** 2025-07-10

**Authors:** Xiangxiong Kong, Terry F. Pettijohn, Hovhannes Torikyan

**Affiliations:** 1 Department of Civil and Geomatics Engineering, California State University, Fresno, 2320 E. San Ramon Ave., M/S EE94, Fresno, CA 93740, USA; 2 Department of Psychology, Coastal Carolina University, P.O. Box 261954, Conway, SC 29528, USA; pettijohn@coastal.edu; 3 Department of Mechanical Engineering, California State University, Fresno, 2320 E. San Ramon Ave., M/S EE94, Fresno, CA 93740, USA; hovhannes@mail.fresnostate.edu

**Keywords:** virtual reality, structural inspection, visual fidelity, photogrammetry, computer vision, civil infrastructure

## Abstract

Civil structures carry significant service loads over long times but are prone to deterioration due to various natural impacts. Traditionally, these structures are inspected in situ by qualified engineers, a method that is high-cost, risky, time-consuming, and prone to error. Recently, researchers have explored innovative practices by using virtual reality (VR) technologies as inspection platforms. Despite such efforts, a critical question remains: can VR models accurately reflect real-world structural conditions? This study presents a comprehensive framework for assessing the visual fidelity of VR models for structural inspection. To make it viable, we first introduce a novel workflow that integrates UAV-based photogrammetry, computer graphics, and web-based VR editing to establish interactive VR user interfaces. We then propose a visual fidelity assessment methodology that quantitatively evaluates the accuracy of the VR models through image alignment, histogram matching, and pixel-level deviation mapping between rendered images from the VR models and UAV-captured images under matched viewpoints. The proposed frameworks are validated using two case studies: a historic stone arch bridge and a campus steel building. Overall, this study contributes to the growing body of knowledge on VR-based structural inspections, providing a foundation for our peers for their further research in this field.

## 1. Introduction

Civil structures such as bridges, buildings, stadiums, tunnels, and dams carry significant service loads over long periods but are prone to deterioration due to various natural impacts such as flooding, humidity, earthquakes, and/or strong winds. According to the American Society of Civil Engineers (ASCE) [1], the bridges in the United States (US) received a grade of C, with 7.5% (or 46,154) of the nation’s bridges being structurally deficient and requiring substantial interventions such as replacements or rehabilitation. In addition, data from the Association of State Dam Safety Officials (ASDSO) [2] indicate that between 2005 and 2013, 173 dam failures and 587 incidents were reported in the US. These figures are significant compared with a total of approximately 92,000 dams in the nation [3]. Therefore, the development of effective structural inspection technologies becomes critical for stakeholders to make appropriate early-stage interventions to prevent catastrophic structural failures.

Traditionally, civil structures are inspected by qualified engineers in situ through field visits. For example, the US Department of Transportation mandates a minimum inspection interval of 24 months for any highway bridges that exceed 9.1 m in length [4], and the Federal Emergency Management Agency (FEMA) in the US provides guidelines for routine dam inspections, which must be performed by qualified engineering professionals [5]. The practice of field inspection, however, is high-cost, time-consuming, and may be prone to inspection error [6,7]. For example, in a study performed by Graybeal et al. [8], inspectors’ skills were assessed using bridges in multiple states in the US. The study reported that only 19 out of 42 inspectors identified a bolt loosening deficiency successfully.

To address these concerns, advanced sensing technologies coupled with unmanned aerial vehicles (UAVs) have shown great promise in civil structure inspections for offering rapid 3D reconstructions of structures [9]. Such high-resolution 3D models contain enriched surface texture information of structure, hence becoming ideal candidates for inspecting structures’ health conditions. By processing the 3D structure model via advanced computational algorithms, the structural deterioration can be identified, quantified, and monitored. Research findings validated that the implementation cost of UAV-based inspection can be dramatically reduced compared to traditional in situ field inspections [10,11].

More recently, researchers [12,13,14,15,16,17,18] have extended UAV-based structural inspection by leveraging virtual reality (VR) technologies as the inspection platforms. This innovation allows users to virtually inspect a digital replica of its physical structure in an office environment. The findings from these studies are highly encouraging, as they verified the technological feasibility of performing structural inspections in a virtual, controlled, and safe in-house environment, compared to field inspections under uncontrolled conditions. As a result, the costly and labor-intensive efforts associated with traditional field inspections are mitigated.

Despite the current efforts, a critical concern is whether or not the established structure models in VR can accurately reflect the real-world conditions of the structures. This concern is legitimate because if there is an inconsistency between an actual structure and its virtual replica in the VR environment, it could lead to incorrect observations and misinformed decisions. For example, a crack in the actual structure may be overlooked by a VR user because it does not appear appropriately in the virtual structure model, or a surface pattern is incorrectly identified as structural damage due to the poor lighting conditions in the VR setup. Therefore, a visual fidelity check of VR models is essential to ensure they accurately represent real-world conditions, minimize observation errors, and enhance the reliability of VR-based inspections.

The primary objective of this study is to propose and validate a framework to assess the visual fidelity of VR models in the context of civil infrastructure inspection. To achieve this, we first present a novel approach to develop VR models of civil structures through photogrammetry reconstruction, computer graphics engines, and an online VR editing platform. Thereafter, we introduce a visual fidelity evaluation framework by analyzing rendered 2D views from VR models and comparing them with UAV-captured images of real structures through image alignment and deviation mapping. These comparisons are made at matched camera positions under the same viewpoint and scene composition. By aligning the camera angles, distances (i.e., focus length), and fields of view between rendered and UAV-captured images, the resulting image pairs enable a consistent assessment of the level of visual fidelity.

The remainder of the manuscript is organized as follows. Section 2 reviews related VR work in structural inspection. Section 3 presents the motivation for this study and identifies the research gap. Section 4 defines the scope of this study. Section 5 outlines the proposed two-phase methodology: Phase I focuses on VR model development, while Phase II introduces a visual fidelity assessment framework of the resulting VR models. Section 6 details the application of our framework to a stone arch bridge, including the complete implementation and evaluation process. Section 7 presents the validation results for a campus steel building, only highlighting key findings due to space limitations. Section 8 further discusses the results, limitations of our study, and future directions. Section 9 concludes the study.

## 2. Related Work

In civil and construction engineering, VR has been proposed for several domains, including construction planning and simulation [19], remote collaboration [20], safety training [21], and design virtualization [22]. Although the concept of VR for structural inspection was introduced more than two decades ago [23], its benefit has only been recognized recently. In this manuscript, we first identified seven relevant studies as shown in Table 1. Our selection criteria are defined as below:

(1) Searching all literature work on Google Scholar [24] and ScienceDirect [25] published since 2017 using keywords “virtual reality” and “structural inspection”.
(2) Excluding studies utilizing augmented reality or mixed reality technologies.
(3) Only considering studies investigating VR models grounded in real-world data of the physical structures.
(4) Excluding studies primarily focusing on VR usability tests.

One of the earliest works in this area was performed by Napolitano et al. [15], who created virtual scenes of a campus pedestrian bridge through 360-degree filming, and integrated sensor networks and monitoring data into a VR user interface. The researchers also collected user feedback on how different groups can effectively collaborate on this inspection project. Later, Omer et al. [12,13] developed VR user interfaces using a 3D scanning device, a smartphone app, and a self-developed software package for bridge inspections. These VR interfaces enabled users to navigate in the virtual environment at their own pace, even accessing bridge locations traditionally inaccessible for inspectors. Attard et al. [16] developed a robotic platform equipped with in situ cameras to film and reconstruct a 3D model of a tunnel. A VR model was then established to visualize a 3D tunnel model, allowing users to view it via VR headsets. Bacco et al. [17] deployed UAVs and photogrammetry to reconstruct 3D models of three historic sites, based on which VR user interfaces were established that allow users to inquire about the data collected from the field sensors. Most recently, Luleci et al. [14] proposed a novel method to fuse structural health monitoring data into a 3D point cloud of a pedestrian bridge, and further visualized it in a VR user interface. The study also demonstrated user interactions within the VR for data checking and decision-making. Lastly, Yiğit and Uysal [18] developed a UAV-photogrammetry workflow that built a high-resolution 3D digital twin of the Elvanlı Bridge, detected cracks in the model, and then streamed the damage-augmented digital twin into a VR platform for inspection.

In terms of VR model establishment, these studies generally employ one of the three approaches below:

(1) The 360-degree camera filming adopted in [15]. Deploying a 360-degree camera in the field is an affordable way to capture the in situ status of a structure. Depending on the structure’s size, multiple 360-degree images shall be collected from various areas of interest. For example, in [15], 27 360-degree images were collected to cover views from both the top and bottom of the bridge. The images were then edited in Photoshop to remove the tripods and correct lighting discrepancies. However, this method has limitations. For large structures, extensive effort would be required to collect enough 360-degree images to cover all areas of interest, especially for inaccessible areas. Also, aligning a large volume of images correctly to form a cohesive VR model could be challenging.
(2) Photogrammetry adopted in [16,17,18]. Built upon Structure-from-Motion and Multi-View Stereo (SfM-MVS) [26,27], photogrammetry is an excellent tool for rapidly reconstructing a 3D model of a structure based on a large volume of 2D digital images taken from different camera positions. In [17], the researchers used a UAV for image collection of historic structures and employed the off-the-shelf software Agisoft Metashape [28] for 3D model reconstruction. In [16], a different platform, 3DFlow Zephyr Aerial [29], was used to reconstruct the 3D point cloud, the mesh, and ultimately the texture mapping model of the tunnel walls.
(3) The 3D LiDAR scanning adopted in [12,13,14], which is another remote sensing method that uses laser light to measure the distances and create a high-fidelity 3D point cloud of a structure [30]. In [14], 11 LiDAR scans were conducted on a 53.9 m long truss bridge to create the 3D point cloud. The data collection process lasted around 5 h and 45 min. In [12,13], researchers adopted Leica ScanStation P40 to establish the 3D point clouds for a masonry bridge and a reinforced box girder concrete bridge. Because raw LiDAR scans have no color information, additional field images were collected and mapped back to the LiDAR point clouds using off-the-shelf capacities embedded in the LiDAR device.

It is worth noting that the VR model development approaches above fundamentally differ from those used to create entirely artificial 3D models, which are common in construction engineering applications such as planning, training, and design visualization [19,21,22]. In those studies, visual fidelity to real-world structures is not a primary concern, allowing for significant alterations to structural texture and/or dimensions. In contrast, for VR models intended for structural inspection, visual accuracy is critical. Regarding VR user interface development, [12,13,14,16,17] utilized Unity [31] to build virtual interfaces; [15] adopted Kolor Panotour Pro [32] to stitch 360-degree scenes together; and [18] used a web-based platform Stratbox VR [33] to create a bridge inspection interface.

In addition to the keyword-based literature search described above, we also conducted a manual citation analysis to enhance the coverage of the relevant literature. This included both backward citation tracking (reviewing the references cited in the studies in Table 1) and forward citation tracking (examining papers that cited those studies in Table 1). Each candidate paper was then manually reviewed to assess whether it met the inclusion criteria defined in this study: investigations of VR for structural inspection that are grounded in real-world structures. This process led to the identification of additional relevant studies, as summarized below.

Several additional studies employed terrestrial laser scanning (TLS) or photogrammetry to reconstruct 3D models for VR-based structural inspection. Savini et al. [34] developed a VR system using laser scans and UAV photogrammetry for bridge inspection, integrating interactive maps, historical data, and online inspection forms. Similarly, Luleci et al. [35,36] combined UAV photogrammetry and LiDAR with health monitoring data and structural analysis to enable collaborative multi-user inspections in a VR environment. Shao et al. [37] constructed a textured VR model of an aging building from TLS data and developed an immersive inspection system with intuitive controls for damage localization and sectional viewing. Ma et al. [38] proposed a panoramic VR interface to visualize real-time monitoring data in a metro tunnel, offering an interactive, web-based inspection tool. Lastly, Fabbrocino et al. [39] developed a web-accessible VR platform using 360-degree virtual tours and interactive maps for the remote inspection and long-term condition tracking of masonry arch bridges.

## 3. Motivation and Research Gap

In VR research, the concept of authenticity has been widely studied, particularly in human–computer interaction, psychology, and training/education. For example, Hameed and Perkis [40] describe authenticity as a sense of trueness and genuineness felt in a virtual place. Gilbert [41] defines it as whether the virtual world can offer the expected experience to the user, both consciously and unconsciously. Wang et al. [42] provide a more detailed framework by defining three interconnected subdomains of authenticity in VR: authenticity of narrative (the storyline and context), authenticity of environment (the sense of presence), and authenticity of action (user tasks and interactions). Despite these discussions, the concepts of authenticity in VR largely depend on user perceptions. Assessing these authenticities typically requires obtaining feedback from the participants through questionnaires or surveys, as users’ subjective experiences and feelings of immersion play a significant role in determining the authenticity of a VR environment.

In contrast, visual fidelity is a technology-centered concept that refers to the extent to which a VR model accurately replicates the surface texture appearance of its real-world counterpart [43]. Unlike authenticity, which depends on how believable a virtual experience feels to users, visual fidelity is an objective property of the digital model itself. This distinction is critical, as it establishes the rationale for evaluating VR models using computational algorithms rather than user testing.

Visual fidelity plays a foundational role in VR-based structural inspections. An inaccurate VR model may distort structural damage (e.g., cracks, corrosion, or material degradation) and lead to incorrect observations. For instance, a structural inspector using a VR model might fail to detect a hairline crack or misinterpret surface rust if the visual representation is not accurately preserved. In this regard, high visual fidelity is a prerequisite for building credible VR user interfaces. Despite the sophistication of the user interface design, if the underlying VR model fails to depict its real-world counterpart, the VR user interface would not yield satisfactory inspection outcomes.

Another important benefit of examining visual fidelity is its potential to support scalable evaluation. Unlike user studies, which can be time-consuming and require participant coordination, computational approaches to fidelity assessment can be applied more efficiently across multiple models and over time as VR content evolves. The visual fidelity evaluation framework proposed in this study may be particularly useful in civil structure asset management, where VR models of various structures could be deployed for inspection across different sites or agencies. In such cases, incorporating a fidelity check could serve as a practical and consistent method for supporting quality assurance.

Despite its importance, visual fidelity assessment remains largely unaddressed in the existing literature. Most prior research reviewed in Section 2 has focused on VR model development [14,18], platform selection [12,13], or user collaborations [15]. While these investigations demonstrated the feasibility of using VR for inspection and explored user interaction techniques, they did not explicitly evaluate how closely the VR models replicate their real-world counterparts. The visual accuracy of model textures was assumed rather than evaluated, leaving a significant gap in the literature. To the best of the authors’ knowledge, no existing study has explicitly addressed the assessment of visual fidelity in VR models for structural inspection.

## 4. Scope of This Study

**Figure 1:**
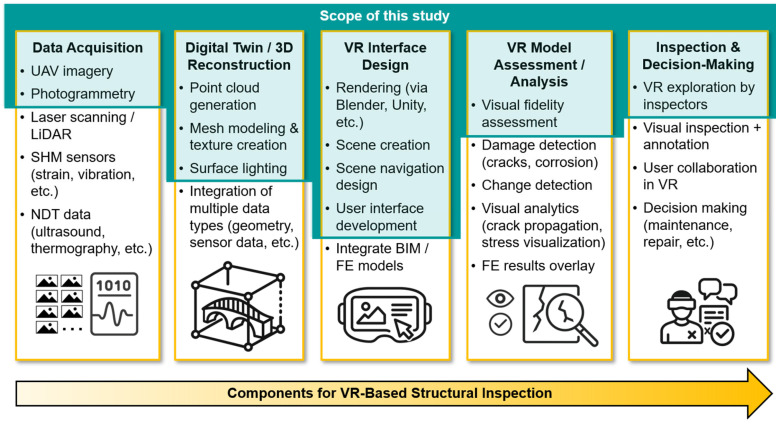
Workflow components of VR-based structural inspection. The scope of this study is highlighted by the teal blue area.

VR-based structural inspection encompasses a multi-component workflow that integrates various technologies for capturing, modeling, visualizing, analyzing, and interpreting the condition of civil infrastructure. As illustrated in Figure 1, a typical VR inspection pipeline includes the following sections: (1) Data Acquisition, where input data such as UAV imagery, photogrammetry, LiDAR scans, structural health monitoring (SHM) sensor measurements, and non-destructive testing (NDT) outputs are collected; (2) Digital Twin/3D Reconstruction, in which point clouds are generated, textured models are created, and various data types are integrated to form high-fidelity digital replicas; (3) VR Interface Design, where rendering, scene creation, and navigation are implemented to enable interactive exploration; (4) VR Model Assessment/Analysis, where visual fidelity shall be evaluated, damage or change is detected, and structural performance can be visualized using overlays such as results from finite element (FE) models; and (5) Inspection and Decision-Making, where inspectors explore the VR environment to perform visual assessments, annotate findings, collaborative tasks, and support maintenance or repair decisions.

This study focuses specifically on frameworks of VR model development and evaluation of visual fidelity of the established models. As highlighted by the teal blue area in Figure 1, our scope includes selected tasks within each of the five workflow components that allow us to directly support our research objective. Tasks falling outside the highlighted area are beyond the scope of our investigation and are therefore not examined. Nevertheless, Section 8.1 and Section 8.3 outline how our proposed framework and findings may still enhance certain tasks that lie outside the scope of this study.

## 5. Methodology

**Figure 2:**
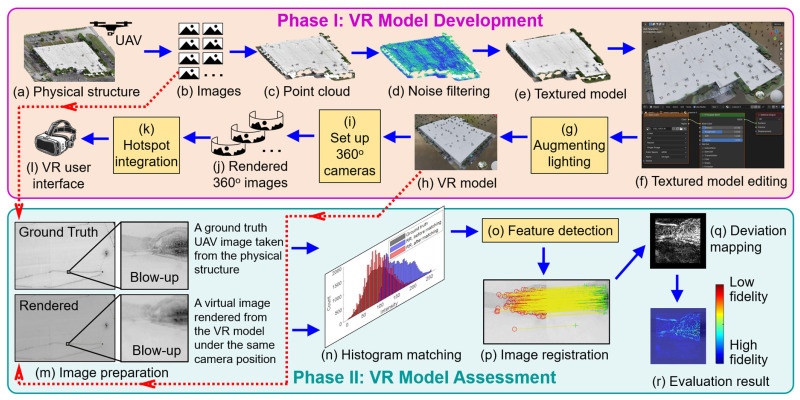
Research methodology overview: Phase I—VR model development, and Phase II—VR model assessment.

Figure 2 illustrates the two-phase research methodology for this study: Phase I involves the development of the VR model, and Phase II focuses on assessing the visual fidelity of the established VR model. Although the primary contribution of this study lies in Phase II, Phase I is essential to enable that process. This is because the evaluation framework in Phase II requires rendered views from a controlled and well-structured VR model, where the camera positions must be known a priori. Existing VR-based structural inspection studies [12,13,14,16,17] rarely release their VR models publicly, and when datasets are available, camera position information may be incomplete. Therefore, Phase I is included to ensure a purpose-built VR model that supports the mission in Phase II. Lastly, to obtain the dataset for developing VR models in Phase I, we selected two testbeds for the validation in this manuscript: a stone arch bridge and a campus warehouse building. These cases were chosen to represent different structural types (bridge vs. building), surface textures, and visual conditions that one would encounter in practice.

### 5.1. VR Model Development

The development of a VR model starts with UAV image collection, as illustrated in Figure 2a. To this end, a UAV flight captures a large volume of high-resolution digital images of the target structure from multiple viewpoints. These images are then processed by a photogrammetry workflow (Figure 2b) to align these UAV images for creating a sparse point cloud, and ultimately generate a 3D dense point cloud of the structure (Figure 2c). Next, a noise removal procedure is conducted to filter out any 3D points with significant reconstruction errors (Figure 2d). Based on the filtered 3D point cloud, a textured model is rendered, which includes both geometric and RGB information about the structure, as shown in Figure 2e.

The established textured model is then imported into a 3D computer graphics engine (Figure 2f) for two main purposes: (1) Enhancing the lighting conditions. A light source is added to the 3D structure model in a virtual environment. The light intensity, light shooting angle, and other parameters are adjusted to optimize the visual clarity of the 3D model. This lighting-augmented textured model is shown in Figure 2h. (2) Rendering the 360-degree images. We set up multiple virtual 360-degree cameras at different locations of the 3D model within the 3D computer graphics engine (Figure 2i). Capturing the structure from different perspectives, these 360-degree images are then rendered (Figure 2j), serving as the immersive virtual scene for establishing the VR user interface.

Next, the rendered 360-degree images are loaded into a VR editing platform to create the VR user interface. This stage involves VR interface design by establishing hotspots in the virtual scenes (Figure 2k), and linking different virtual scenes into a cohesive VR unit through transition hotspots. Finally, the established VR interface is tested on the VR editing platform and then loaded onto the VR headset, allowing the user to interactively explore the structure in a fully immersive virtual environment (Figure 2l).

### 5.2. VR Model Assessment

To assess the visual fidelity of the VR model, the first step is to identify two image sources for comparison. These include (1) a UAV-captured image of the structure obtained during the initial image collection, considered as the ground truth (Figure 2b); and (2) a rendered image of the VR model under the same camera position, as illustrated in Figure 2h. Because the UAV camera positions are reserved during Phase I, the rendered image can be produced from the same viewpoint in the computer graphics engine as the field-captured ground truth UAV image. An example pair of ground truth and rendered images is shown in Figure 2m.

Due to minor reconstruction inaccuracies during the photogrammetry process in Phase I, the ground truth and rendered images may not initially align perfectly. To address this, we employ an image registration approach, which begins by aligning the rendered image to the coordinate system of the ground truth image. This stage includes detecting feature points from both images (Figure 2o), computing a geometric transformation matrix, and applying the matrix to the rendered image, as shown in Figure 2p. As a result, a recovered rendered image that fully aligns with the ground truth image is produced. Depending on the rendering configuration and lighting conditions implemented in Phase I, histogram matching may also be applied to normalize the intensity distribution before image registration to ensure consistent visual comparison (Figure 2n).

Next, the absolute intensity differences between the ground truth and recovered rendered images are subtracted using their grayscale first, as illustrated in Figure 2q. These differences are then converted into an RGB scale (heatmap), where blue indicates minimal differences (i.e., high visual fidelity) and red indicates large discrepancies (i.e., lower visual fidelity). Finally, to support intuitive interpretation, the RGB heatmap is overlaid onto the original ground truth image patch, producing a composite visualization as shown in Figure 2r.

## 6. Bridge Validation

**Figure 3:**
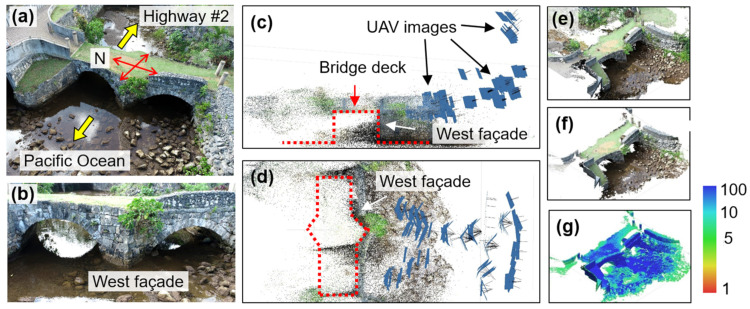
(**a**,**b**) present the bird’s eye view and west façade views of the stone arch bridge, respectively. (**c**,**d**) illustrate the UAV camera positions (i.e., small blue patches) in elevation and plan views, with red dashed lines indicating the bridge outline. (**e**) displays the initial dense point cloud; (**f**) shows the final dense point cloud after filtering out low-confidence points; (**g**) displays a color plot of the final dense point cloud based on the point confidence distribution.

The Taleyfac Spanish Bridge on the island of Guam, a US territory in the Western Pacific, is adopted for this validation. The bridge was originally constructed in 1785 as a wooden bridge, replaced by a stone arch bridge in 1866 [44,45]. After being governed by the US Navy for transportation until 1917, the bridge became obsolete and gradually faced deterioration. Over time, it suffered damage, including a missing portion of the south arch, as well as impacts from floods, storms, and earthquakes [44]. In 2013, the bridge was restored with the support of the Guam Preservation Trust. Figure 3a,b illustrate the bridge, which is located on the west side of the island, close to Guam Highway #2. The bridge has a length of 10.7 m and can be accessed through the platforms on both the north and south ends, spanning over a stream that flows toward the Pacific Ocean.

### 6.1. VR Model Development

#### 6.1.1. Point Cloud Reconstruction via SfM-MVS

To reconstruct the 3D point cloud of the bridge, we employed a DJI Phantom 4 Pro+ V2.0 (DJI Sky City, Shenzhen, China) to capture UAV images in the field. The UAV was programmed to automatically take images at a fixed time interval of 2 s. The west façade (defined in Figure 3d) was selected as the target area for bridge reconstruction in this validation. A total of 193 UAV images were collected, each taken from different elevations, shooting distances, and camera angles, as indicated by the small blue patches in Figure 3c,d. Adjacent UAV images usually had a significant overlap between 70% and 80% and are under a local coordinate system without georeferencing. For a detailed discussion on the field image collection of the bridge, the readers are referred to [46].

Once obtained, the field UAV images were processed using Agisoft Metashape (Professional Edition, version 1.6.2) [28], an off-the-shelf photogrammetry tool that has been widely applied in various engineering fields [47,48,49]. To this end, UAV images were initially aligned through SfM-MVS [26,27] to generate the sparse point cloud. Thereafter, a dense point cloud of the west façade was reconstructed, consisting of 9.3 million 3D points (Figure 3e). To further refine the data, points located far from the camera positions, such as trees, bridge platforms, and the backside (i.e., east façade) of the bridge, were removed. Next, point confidence, a unitless parameter that presents the reconstruction error with a range from 0 to 100, was computed for all 3D points. Points with confidence below 5 were filtered out due to large reconstruction errors, leading to a truncated dense point cloud consisting of 2.6 million 3D points (Figure 3f). The color plot in Figure 3g shows the same point cloud as Figure 3f but depicts the distribution of the point confidence, where low-confidence points ranging from red to yellow were eliminated.

#### 6.1.2. Textured Model Generation

**Figure 4:**
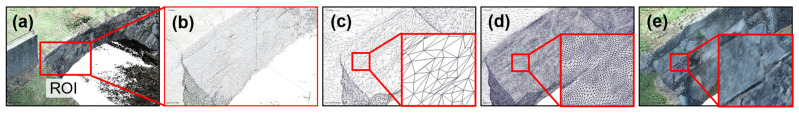
(**a**–**e**) illustrate the process of textured model creation: (**a**) ROI selection; (**b**) dense point cloud within ROI; (**c**) initial wireframe model before mesh refinement; (**d**) refined wireframe model; and (**e**) final textured model.

Following the dense point cloud of the bridge’s west façade, we proceeded with creating the wireframe (i.e., mesh) model in Agisoft Metashape. To better illustrate the result, a region of interest (ROI) is defined as a small area on the west façade (see the red rectangular box in Figure 4a). The blow-up view of the dense point cloud within the ROI is shown in Figure 4b, and the established wireframe model under the same area is shown in Figure 4c. To achieve a finer mesh distribution, we utilized the auto-mesh-refining function to update the initial mesh. The refined wireframe results are shown in Figure 4d. As can be seen from the figures, the refined mesh model in Figure 4d can more accurately depict the geometric shapes of the bridge surface compared with the results in Figure 4c. Finally, based on the refined wireframe model, the texture of the bridge model was rendered, as depicted in Figure 4e.

#### 6.1.3. Texture Augmentation

**Figure 5:**
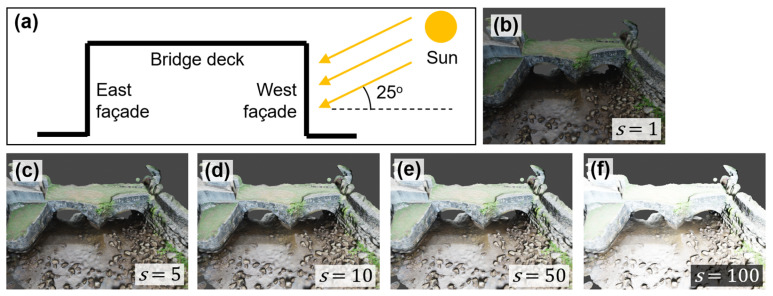
(**a**) presents a schematic illustrating the artificial sunlight simulation setup; (**b**–**f**) show the results of the lighting simulation under different sun strength values, *s*.

We augmented the texture of the bridge model by establishing artificial lighting conditions via a computer graphics engine, Blender [50]. To elaborate, the textured model was first exported from Agisoft Metashape as two files: a DAE (i.e., COLLADA) model containing the geometric features of the bridge without colors, and an image (TIFF) file representing high-resolution RGB information of the bridge’s surface. Subsequently, both files were imported into Blender, and the RGB information was mapped back to the DAE model. To configure the artificial lighting conditions, we utilized sunlight as the light source after exploring various light options in Blender. Sunlight was deemed more suitable for simulating outdoor lighting compared with other light sources such as point, spot, or area lights. The sunlight angle was set at approximately 25 degrees, as shown in Figure 5a. The specific location of the sun would not affect the simulation results, as all sunlight beams were parallel and the actual location of the sun was considered infinitely distant from the bridge, regardless of the selected sun’s location.

After determining the sunlight angle, we further tuned the sun strength parameter, denoted as *s*. Ranging from 0 to 1000, this parameter represents the intensity of sunlight. Figure 5b to f illustrate the simulation results under different values of *s*. As can be seen from Figure 5b, a low value of *s* (*s* = 1) causes insufficient lighting conditions on the bridge. This may negatively affect the performance of structural inspection in the virtual environment. On the other hand, a high value (*s* = 100, Figure 5f) leads to overexposure of the bridge model. In this case, useful information about the bridge’s surface may be difficult to see. To achieve an optimal sunlight effect, we adopted *s* = 10 as the final choice.

Notice that through the photogrammetry workflow, the textured model of the bridge inherently contains original lighting conditions from the field. For example, the presence of shadows caused by the in situ sunlight cannot be completely removed by artificial sunlight. In this regard, the simulations of the artificial sunlight in Blender only serve as the means to augment the lighting conditions of the bridge rather than completely override field lighting conditions.

#### 6.1.4. VR Scene Creation

**Figure 6:**
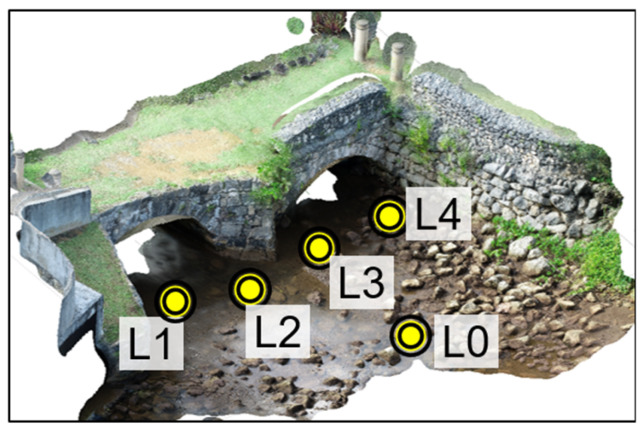
360-degree camera locations implemented in Blender, indicated by yellow dots.

To create the VR scene, we established a virtual equirectangular-panoramic-style 360-degree camera in Blender. The camera was positioned horizontally to replicate a human field of view. Then, the 360-degree camera was placed at five different locations, as shown in yellow dots in Figure 6. Among these, L0 was in front of the bridge’s west façade, while L1, L2, L3, and L4 represented four camera locations close to the stone surfaces. Later on, rendered images at these locations will be linked together to create the VR user interface. The 360-degree images were rendered at each of the five camera locations with an image resolution of 2000 × 1000 pixels.

#### 6.1.5. VR User Interface

**Figure 7:**
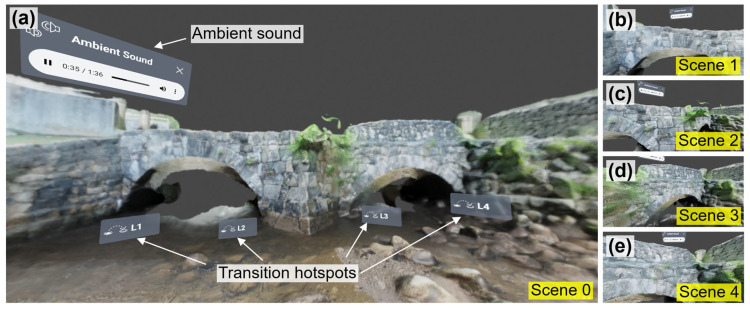
(**a**–**e**) Screenshots of the VR user interface taken from a computer monitor.

We adopted VIAR360 [51], an online web-browser-based VR editing platform, to build the VR user interface. Table 2 offers an overview of how different media components were integrated into the final VR user interface. Figure 7 illustrates the screenshots of five scenes of the final VR user interface. These screenshots are directly captured from the VIAR360 editing interface via a web browser on a computer monitor. Our previous studies [52,53] offer detailed discussions on the capacities of VIAR360 for VR interface establishments.

As can be found in Figure 7, a total of five VR scenes, denoted Scene 0 to Scene 4, were first created based on the rendered 360-degree images at the camera locations in Figure 6. Among these, Scene 0 serves as the first scene where the users start their journeys, offering an overview of the bridge’s west façade from a point of view located in the middle of the stream (Figure 7a). The remaining VR scenes (i.e., Scenes 1 to 4) provide close-up views of different areas, representing the detailed surface conditions of the bridge (see Figure 7b–e). For instance, Scenes 1 and 2 are close to the stone arch on the north; while Scenes 3 and 4 depict the arch on the south.

As can be found in Table 2, the following features were added to each VR scene. These features include (1) the designed point of view to govern the users’ initial viewing perspective upon entering the scene; and (2) a copyright-free ambient sound [54] to establish a relaxed atmosphere for users to explore the bridge structure. This ambient sound, as illustrated in Figure 7, can be adjusted or muted according to the user’s preference. To enhance the interactivity of the VR user interface, transition hotspots were added to each VR scene, as explained in the last column of the table. These transition hotspots enable navigation between different VR scenes. For example, a user can select a route as Scene 0 → Scene 1 → Scene 2 → Scene 3 → Scene 4 to complete the inspection. Alternatively, the user may elect Scene 0 → Scene 4 → Scene 3 → Scene 2 → Scene 1. Figure 7a shows the callouts of transition hotspots that allow the user to visit Scenes 1 to 4, while the transition hotspots are also added to other VR scenes, but are outside the screenshots in Figure 7b–e.

#### 6.1.6. Implementation via VR Headset

**Figure 8:**
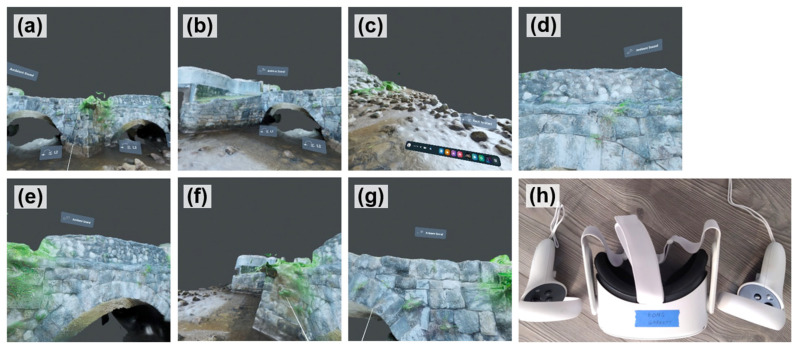
(**a**–**g**) Screenshots taken from the VR headset; (**h**) the Oculus (Meta) Quest 2.

To implement the established user interface in the VR headset, we utilized Oculus Quest 2, now rebranded as the Meta Quest 2 [55] (Meta Quest 2 thereafter), shown in Figure 8h. The established VR user interface is run through the VIAR360 Virtual Player, a VR app that can be installed via the Meta Quest Store. Figure 8a–g are screenshots of the VR user interface randomly taken from Meta Quest 2 from the first-person perspective under different scenes. As can be seen from the figures, a user can navigate to different areas of the bridge, inspecting the bridge façade from different perspectives.

### 6.2. VR Model Assessment

**Figure 9:**
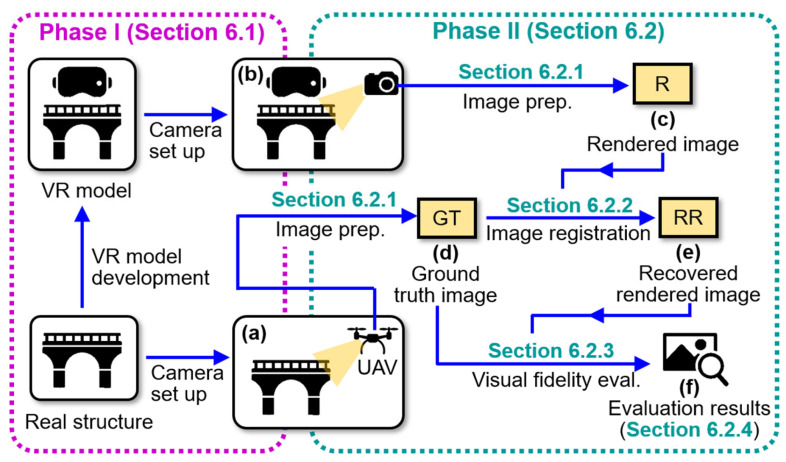
Schematic illustrating the procedure for assessing the visual fidelity of the VR model: (**a**) camera setup in the real structure; (**b**) camera setup in the VR model; (**c**) Blender-rendered image; (**d**) ground truth image; (**e**) recovered rendered image; and (**f**) results of visual fidelity assessment. GT = ground truth; R = rendered; RR = recovered rendered.

Our goal for VR model assessment is to investigate the extent to which the bridge VR model accurately reflects its physical counterpart in the real world. Figure 9 overviews the two-phase procedure, where Phase I has been discussed in Section 6.1. To begin Phase II, we first set up the cameras in both the real structure and the VR model, as shown in Figure 9a and b. Then, the rendered image from the VR model (Figure 9c) and a ground truth image from the UAV (Figure 9d) are obtained, both under the same camera position. Section 6.2.1 discusses how to prepare these images. Next, these two images are aligned through an image registration procedure to produce a recovered rendered image (Figure 9e), as explained in Section 6.2.2. In Section 6.2.3, we assess visual fidelity by comparing the newly generated recovered rendered image with the ground truth image using deviation mapping. Lastly, Section 6.2.4 presents the evaluation results (Figure 9f). To avoid ambiguity in terminology and ensure clarity in the discussion of this subsection, Table 3 summarizes the key items used in our VR model assessment.

#### 6.2.1. Image Preparation

**Figure 10:**
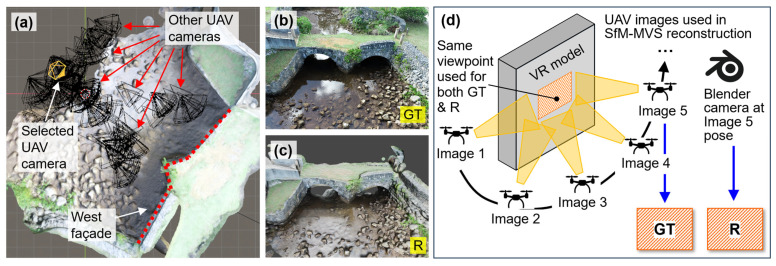
(**a**) Blender interface showing the selected UAV camera position for rendering; (**b**) UAV-captured ground truth image from the selected camera position in (**a**); (**c**) rendered image from the selected camera position in (**a**); (**d**) a schematic showing the visual fidelity assessment using a matched camera view. GT = ground truth; R = rendered.

To prepare the rendered image, we revisited the VR model established in Phase I in Blender. Figure 10a illustrates the Blender interface with all UAV camera positions imported from the photogrammetry workflow (presented by black cones). Next, for illustration purposes, one UAV camera position was randomly selected, highlighted by the orange cone in the figure. To ensure a fair comparison between the rendered and ground truth UAV images, the Blender camera was configured with a resolution of 4864 × 3648 pixels, matching the specifications of the UAV images. The focal length was manually adjusted to approximate the same depth of the UAV image, with a value of 26 mm. The rendered image, produced by Blender’s rendering engine, is shown in Figure 10c. The corresponding UAV image is shown in Figure 10b and serves as ground truth. These two images are the inputs for the processing work explained in the following subsections. Using a matched camera pose for both ground truth and rendered images enables a visual fidelity assessment in a controlled viewpoint. This eliminates perspective change in field of view, while ensuring that any observed discrepancies can be attributed solely to VR modeling limitations.

It is important to note that although the rendered image and the UAV-captured ground truth image are generated from the same camera viewpoint, the rendered image is not produced directly from that single UAV image. Rather, it is synthesized in Blender using a virtual camera projected onto the VR model reconstructed via SfM-MVS. Such a VR model is built from many overlapping UAV images that collectively cover the same region. Figure 10d illustrates this concept: a selected camera viewpoint (orange area) in the VR model captures a structural surface that has been reconstructed using visual and geometric information from five overlapping UAV images. Therefore, the rendered image generated from the camera position corresponding to Image 5 represents a composite synthesis of the model, not a replication of any individual UAV image, including the one from the matched viewpoint (i.e., Image 5). Thus, the rendered image in Figure 10c does not originate from the matched UAV image shown in Figure 10b, but instead reflects a synthesized projection from the photogrammetric model.

#### 6.2.2. Image Registration

The ground truth and rendered images (Figure 10b,c) may exhibit slight misalignment in their perspective views due to two factors: (1) errors induced during the estimation of camera position using SfM-MVS; and (2) the error from manual focal length adjustment, which affects the scale of the image scene. To ensure an image comparison under a consistent view, it is necessary to align the ground truth and rendered images into the same image coordinate system. To achieve this, a series of algorithms were employed via MATLAB Computer Vision Toolbox [56]. These algorithms perform feature matching and geometric transformation to register the rendered image with the ground truth image, and can further minimize the disparities between them.

**Figure 11:**
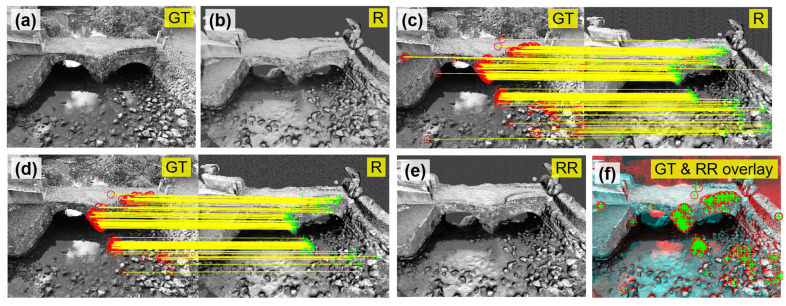
(**a**) Grayscale ground truth image; (**b**) grayscale rendered image; (**c**) initial feature matching results between (**a**) and (**b**); (**d**) final feature matching results after eliminating outliers; (**e**) recovered rendered image after applying geometric transformation to (**b**); and (**f**) overlay of (**a**) and (**e**). Red circles and green crosses are features detected from different images, respectively. GT = ground truth; R = rendered; RR = recovered rendered.

To explain, we first converted both ground truth and rendered images from RGB to grayscale (see Figure 11a,b). Then, Shi-Tomasi [57] feature points were extracted from these grayscale images. Feature points are localized patches with unique intensity distributions, which are invariant to image scale changes, allowing them to be consistently detected across images captured from different sources. Although for this study, Shi-Tomasi [57] features were used; other feature detectors such as BRISK [58], SIFT [59], Harris–Stephens [60] can also be used for similar purposes. For more explanation of feature points, readers are referred to [61,62].

To implement feature tracking, we employed the Kanade–Lucas–Tomasi (KLT) tracker [63,64]. The tracking results as shown in Figure 11c where red circles are features extracted from the ground truth image, and green crosses are those detected in the rendered image. Notice that at this stage, the feature-tracking results may contain incorrect pairings (i.e., outliers). To further enhance tracking accuracy, we computed a projective geometric transformation matrix [27] based on all tracked feature pairs. This matrix depicts the spatial relationship between image coordinates in the two views. Feature points that adhered to this transformation are considered inliers, while those that deviated are classified as outliers. Figure 11d shows the filtered matching results (i.e., inliers) after eliminating outliers. It is important to note that the accuracy of the projective transformation depends on the distribution of feature points across both ground truth and Blender-rendered images. To ensure an accurate transformation matrix, we verified that the matched feature points were well-distributed over the images before applying the transformation.

Next, we applied the established transformation matrix to the rendered image (Figure 11b), producing a new image, noted as the recovered rendered image in Figure 11e. This recovered image has a slightly adjusted viewpoint compared to the original rendered image (Figure 11b), but is now fully aligned with the perspective of the ground truth image (Figure 11a). In Figure 11f, we present an overlay of the ground truth and recovered rendered images, where red circles and green crosses are well aligned across the image, indicating that these two images are fully registered together.

#### 6.2.3. Visual Fidelity Evaluation

**Figure 12:**
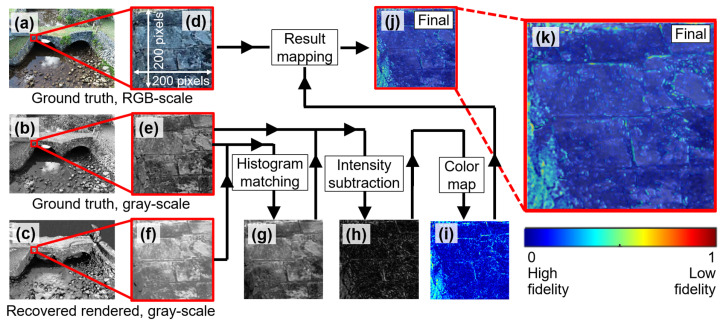
Schematic of visual fidelity evaluation: (**a**) ground truth image; (**b**) grayscale ground truth image; (**c**) grayscale recovered render image; (**d**) image patch from (**a**); (**e**) image patch from (**b**); (**f**) image patch from (**c**); (**g**) recovered rendered image after histogram matching; (**h**) absolute intensity subtraction between (**e**) and (**g**); (**i**) color-coded heatmap; (**j**) overlap of (**d**) and (**i**); (**k**) the magnified view of (**j**).

Figure 12 shows the workflow to evaluate the visual fidelity of the VR model. To start, both RGB and grayscale ground truth images were first selected, as shown in Figure 12a,b. In addition, the grayscale recovered rendered image, obtained through discussion earlier, was prepared (Figure 12c). Since our study area focused on the west façade of the bridge, we specifically identified a localized image patch measuring 200 × 200 pixels. The corresponding patches under this small area are shown in Figure 12d–f. These patches defined the ROI used for the subsequent visual fidelity analysis.

Next, our focus shifted to the grayscale ground truth and recovered rendered patches (Figure 12e,f). Notably, these two patches exhibited different brightness levels, which posed challenges in evaluating their visual similarity. To address this issue, we applied histogram matching to the recovered rendered patch (Figure 12f), aligning its histogram with that of the ground truth patch (Figure 12e). The recovered rendered patch after this adjustment is shown in Figure 12g. The histogram matching ensures that observed deviations more accurately reflect structural differences rather than lighting variation.

**Figure 13:**
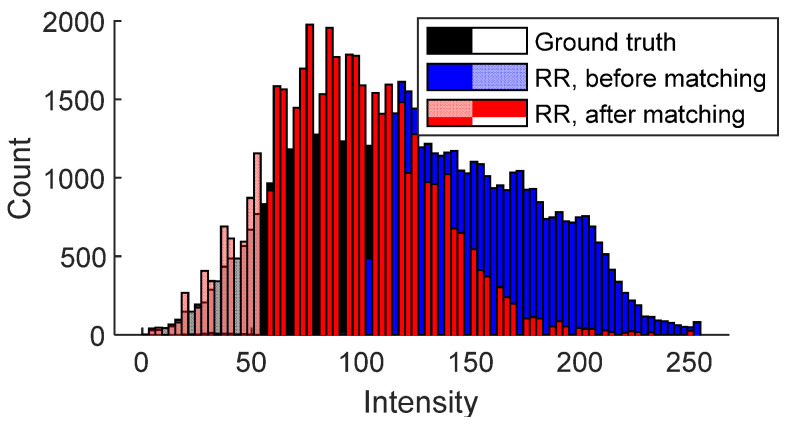
Histograms of the ground truth image patch, the RR image patch before histogram matching, and the RR image patch after histogram matching. RR = recovered rendered.

To further illustrate the concept of histogram matching, Figure 13 compares histograms of the ground truth patch (Figure 12e), the recovered rendered patch before histogram matching (Figure 12f), and the recovered rendered patch after histogram matching (Figure 12g). As can be seen from the figure, the blue histogram (original recovered rendered patch) is adjusted to align with the black histogram (ground truth patch), leading to the red histogram (updated recovered rendered patch after matching). As a result, the adjusted recovered rendered patch shared a similar brightness with the ground truth patch (see black and red histograms).

Next, we performed a series of image processing steps, as illustrated in Figure 12h–k. To explain, let $I_{GT}$ denote the grayscale ground truth image patch (Figure 12e) and $I_{RR}$ denote the histogram-matched recovered rendered patch (Figure 12g). We first computed the absolute intensity difference at each pixel (x,y) as

(1) $$D\left(x,y\right)=\left|{I_{GT}\left(x,y\right)-I}_{RR}(x,y)\;\right|$$

The resulting matrix D represents a per-pixel deviation map with intensity values in the range [0, 255], where a value of 0 (black) indicates perfect visual agreement and a value of 255 (white) indicates maximum mismatch. Gray levels in between (intensity from 1 to 255) represent some extent of unmatched pixel intensity. An example of this grayscale deviation map is shown in Figure 12h.

To enhance interpretability, the deviation matrix D was normalized to the range [0, 1] using

(2) $$D_{norm}=(D-min(D))/(max(D)-min(D))$$

The normalized matrix $D_{norm}$ was then mapped to an RGB heatmap using the jet colormap (Figure 12i). This color mapping assigns blue to areas of low deviation (i.e., high visual fidelity), red to areas of high deviation (i.e., low visual fidelity), and intermediate colors such as cyan and yellow to represent varying levels of difference in between.

The resulting RGB heatmap (Figure 12i) was overlaid onto the original RGB ground truth patch (Figure 12d) using alpha blending with 50% transparency. This allows the user to see both the underlying image structure and the color-coded intensity mismatches simultaneously. A colorbar was included to indicate the full scale of deviation values. The final composite visualization is shown in Figure 12j, with a zoomed-in view provided in Figure 12k. Together, these overlaid visual outputs offer an intuitive and spatially precise understanding of where the VR model maintains fidelity and where discrepancies occur relative to the ground truth.

In this study, we intentionally avoid using conventional image similarity metrics such as structural similarity (SSIM) [65], peak signal-to-noise ratio (PSNR) [66], or feature similarity (FSIM) [67] for visual fidelity evaluation. While these metrics are widely used for pixel-level comparisons in image restoration or compression tasks, they assume that both images originate from nearly identical sources and are pixel-wise aligned. In our case, the rendered images are synthesized through SfM-MVS reconstruction, while the ground truth images are captured directly from a UAV. Despite careful viewpoint matching and image registration, the inherent differences in source modalities would make these metrics less reliable. Instead, we adopt a deviation mapping approach, which provides localized, interpretable results. This method enables the detection of fine-scale surface discrepancies that may not be captured by global similarity scores. For a detailed discussion, readers are referred to Section 8.1.

#### 6.2.4. Evaluation Results

**Figure 14:**
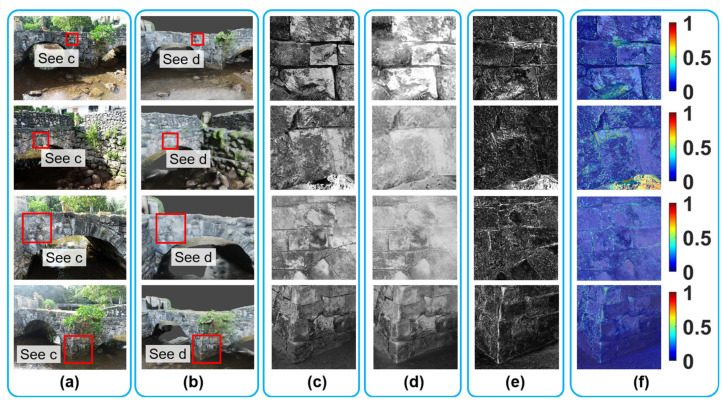
Evaluation results of four different camera positions: (**a**) ground truth UAV images; (**b**) corresponding rendered images; (**c**) grayscale ground truth image patches cropped from ground truth UAV images; (**d**) grayscale patches cropped from recovered rendered images; (**e**) absolute pixel-wise comparison between (**c**) and (**d**); (**f**) final evaluation results with color plots superimposed on the ground truth patches.

Following the protocol explained above, we expanded our investigation to assess the visual fidelity of the VR model across 12 different locations. Due to the limited space, only results from four of them are shown in this subsection (Figure 14), while the results for the remaining eight bridge locations can be found in Appendix A, Figure 14a,b display the ground truth images captured from selected camera angles, along with their corresponding rendered images.

To further process the data, small red boxes were defined in Figure 14a,b to quantify regions of interest on the bridge’s façade. The corresponding grayscale patches extracted from the ground truth images are shown in Figure 14c, while those from the recovered rendered images are presented in Figure 14d. Due to space constraints, the full views of the recovered rendered images are not included in the figure. Thereafter, the image registration and evaluation protocol was applied, including histogram matching, geometric transformation, and deviation mapping. The results of pixel-wise intensity subtractions between and ground truth and recovered rendered patches are shown in Figure 14e. These results were then converted into heatmap representations, as shown in Figure 14f.

**Figure A1:**
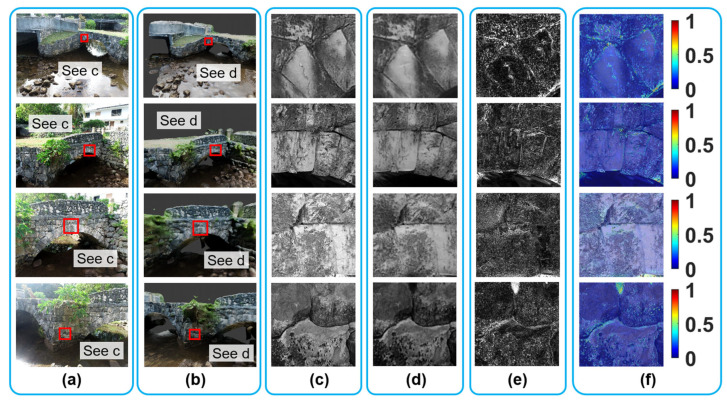
Evaluation results of four different camera positions: (**a**) ground truth UAV images; (**b**) corresponding rendered images; (**c**) grayscale ground truth image patches cropped from ground truth UAV images; (**d**) grayscale patches cropped from recovered rendered images; (**e**) absolute pixel-wise comparison between (**c**) and (**d**); (**f**) final evaluation results with color plots superimposed on the ground truth patches.

**Figure A2:**
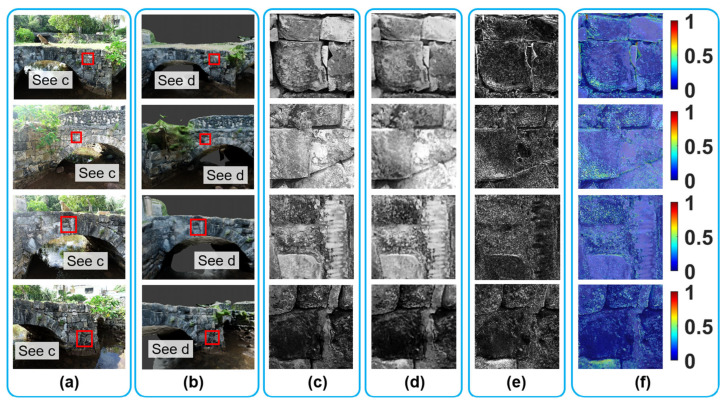
Evaluation results of four different camera positions: (**a**) ground truth UAV images; (**b**) corresponding rendered images; (**c**) grayscale ground truth image patches cropped from ground truth UAV images; (**d**) grayscale patches cropped from recovered rendered images; (**e**) absolute pixel-wise comparison between (**c**) and (**d**); (**f**) final evaluation results with color plots superimposed on the ground truth patches.

The assessment results shown in Figure 14, along with the remaining results in Figure A1 and Figure A2 (Appendix A), collectively demonstrate that the VR model of the bridge exhibits an overall high level of visual fidelity in replicating the structural conditions observed from field UAV images, regardless of different assessment places across the bridge façade. This suggests that the texture information rendered in the VR model closely reflects the accurate condition of the bridge in the real world, providing users with an accurate representation of the bridge’s surface details. However, certain areas of medium to low visual fidelity are observed, indicated as brighter regions in yellow and red in Column f. These discrepancies arise from two main factors: (1) inaccuracies of the VR model’s environment, such as the VR model’s failure to accurately reconstruct the stream beneath the bridge, as observed in the lower right corner of the second row in Figure 14f; (2) residual image registration errors, where minor misalignments persist despite the geometric transformation, leading to localized mismatches, such as the vertical white line as shown in the fourth row of Figure 14f. Additional discussion on the sources of error can be found in Section 8.1.

## 7. Building Validation

To investigate the robustness of the proposed methodology, we selected a single-story steel warehouse building as the testbed for the second validation. Our focus is on the roof area, where a VR user interface was developed for structural inspection. As the technical procedures of our proposed framework have been thoroughly detailed in Section 6, this section only briefly presents the key interim and final results.

**Figure 15:**
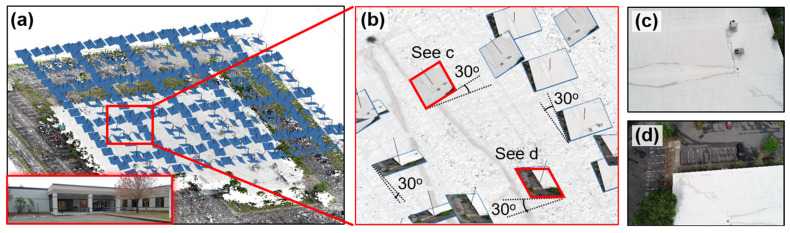
(**a**) UAV camera positions over CSC (i.e., small blue patches); (**b**) a magnified view of UAV camera positions; (**c**,**d**) sample UAV images collected in the field.

The selected building is the Coastal Science Center (CSC) at Coastal Carolina University, located in Conway, South Carolina, in the US. Originally constructed in 1998 by a private owner, the CSC building is a single-story steel warehouse structure that was acquired by the university in the early 2000s. As shown in the lower left corner in Figure 15a, the building served as classrooms, laboratories, and offices, primarily for the Gupta College of Science. With a floor plan measuring 97.5 m by 70.0 m, the CSC features a roof structure system consisting of pre-fabricated steel trusses and wide rib steel roof decks. On the architectural side, tapered rigid insulation is installed on the steel roof decks, on top of which a rubber-like white material, Ethylene Propylene Diene Monomer (EPDM) is installed to provide weatherproof protection for the CSC building.

### 7.1. VR Model Development

To collect UAV images of the CSC building, we used a DJI Phantom 4 Pro and planned the flight route via Pix4Dcapture [68]. The flight mission employed a double grid flight mode [69] with 90% front overlap and 75% side overlap, allowing the UAV to automatically take images along the pre-defined grid lines (see Figure 15a). A total of 342 images were collected in the field. Instead of pointing directly downward, the UAV camera was tilted at a 30 degree angle, as shown in Figure 15b to enhance reconstruction results. Two representative UAV images, highlighted in red diamond boxes in Figure 15b, are shown in Figure 15c,d.

**Figure 16:**
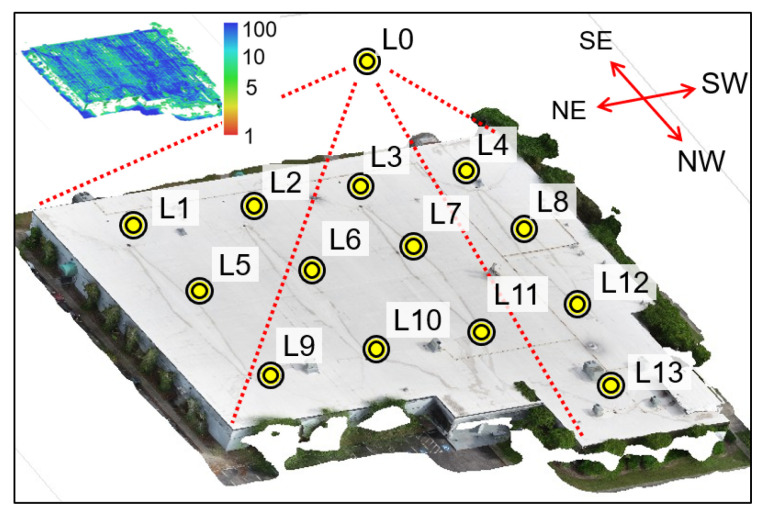
Textured model of CSC. Yellow dots are 360-degree camera locations. The color plot against the point confidence is shown in the top-left corner.

The collected UAV images were processed using SfM-MVS in Agisoft Metashape to generate a series of results, including sparse point cloud, dense point cloud, wireframe model, and textured model. Due to the limited space, only the textured model is shown in Figure 16. Initially, the dense point cloud contained 29.1 million 3D points. To refine the results, we removed 3D points in the parking areas as they were outside the scope of this study. In addition, the 3D points with point confidences below 5 were filtered out, leaving the final dense point cloud containing 6.5 million points. The color plot of the filtered dense point cloud is illustrated in the top-left corner of Figure 16 where low confidence points, ranging from red to yellow, were eliminated from the results. Because the scope of this validation focuses on the building’s roof only, no UAV images were taken around the building facades. Therefore, the elevations of the building may contain reconstruction gaps, as can be seen in the NW façade in Figure 16.

Next, the textured model of the CSC building was imported into Blender for further adjustments, including tuning the sunlight strength and defining the camera position for rendering 360-degree images. Since the roof surface is predominantly white, a lower sunlight strength value (s = 2) was applied to reduce glare and enhance the visibility of roof details (e.g., water stains). To further optimize lighting, the sunlight angle was set at 90 degrees to maximize the sunlight effect on the roof. A total of 14 360-degree camera positions were defined in Blender, as shown in the yellow dots of Figure 16. Among them, camera position L0 was positioned on the top of the entire roof to offer an overview of the roof, while camera positions L1 to L13 were placed near specific roof sections to provide detailed views.

**Figure 17:**
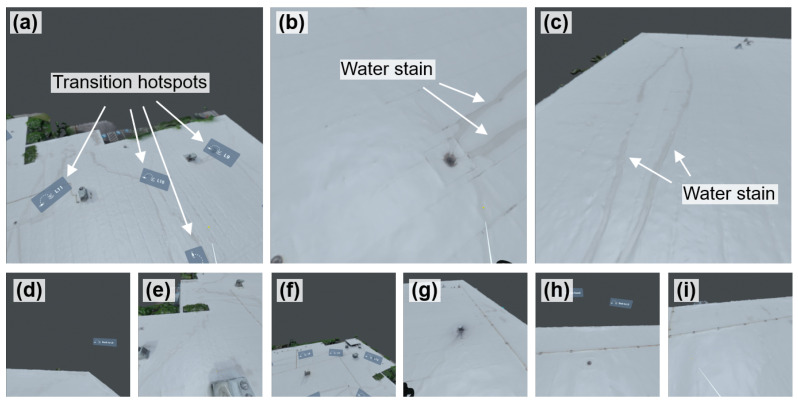
(**a**–**i**) Screenshots of the VR user interface taken from the VR headset.

Next, the rendered 360-degree images at these camera positions were integrated into the VR user interface via VIAR360. A total of 14 VR scenes, labeled Scenes 0 to 13, were established using the images rendered from camera positions L0 to L13. Each scene was configured with a predefined point of view to control the user’s initial orientation when entering the scene. The ambient sound used in bridge validation [54] was reused across all virtual scenes in this case study. Scene 0 served as the entry point of the VR experience, allowing users to begin with an overview of the entire roof area. Multiple transition hotspots were embedded in Scene 0 to allow the user to navigate to other scenes (i.e., Scenes 1 to 13), which offer close-up views of specific roof sections. Figure 17a illustrates some of these transition hotspots. Additionally, a return hotspot linking back to Scene 0 was added in each of the subsequent scenes.

Figure 17 shows the screenshots taken from the VR user interface of the Meta Quest 2 headset. As shown, users can select different areas to explore by clicking on the hotspots (Figure 17a). This interactive design enables the user to inspect detailed features of the roof surface, including visible elements such as water stains (Figure 17b,c). Figure 17d–i show other screenshots from the VR user interface from the user’s perspective.

### 7.2. VR Model Assessment

**Figure 18:**
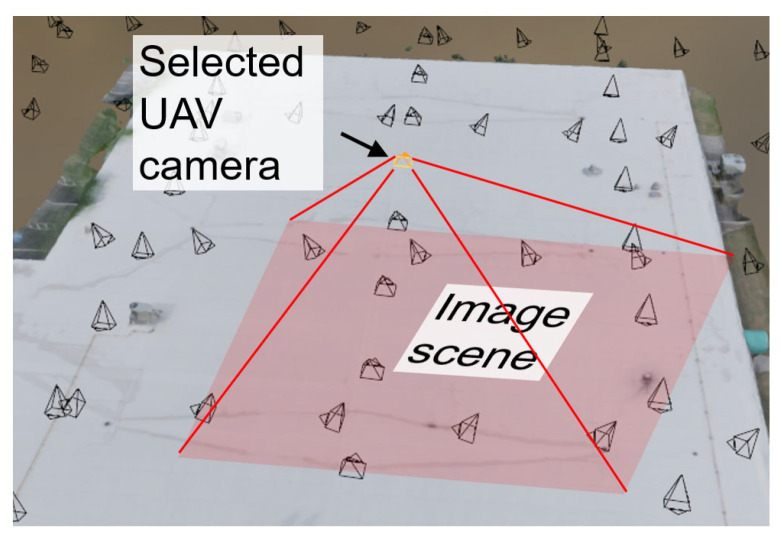
Image rendering setup in Blender.

**Figure 19:**
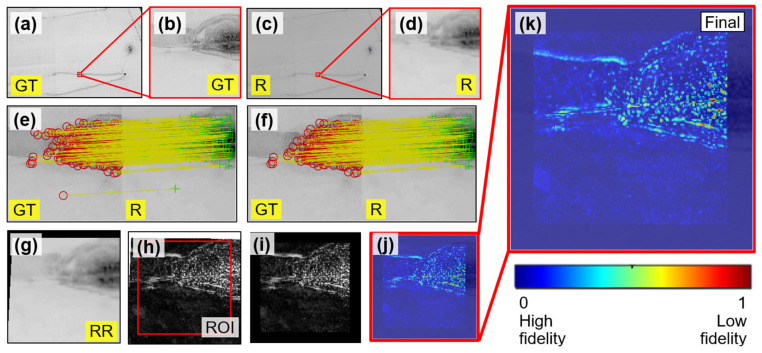
Updated visual fidelity evaluation protocol: (**a**) ground truth image; (**b**) image patch from (**a**); (**c**) Blender-rendered image; (**d**) image patch from (**c**); (**e**) initial feature matching between (**b**) and (**d**); (**f**) feature matching after eliminating outliers; (**g**) recovered rendered image patch; (**h**) absolute intensity subtraction between (**b**) and (**g**); (**i**) result after applying a mask and assign black color outside the ROI in (**h**); (**j**) final visual fidelity assessment result; and (**k**) the magnified view of (**j**). GT = ground truth; R = rendered; RR = recovered rendered.

The texture features of the building’s roof differ from the bridge’s façade discussed in the previous validation. Specifically, the roof areas are predominantly white and contain fewer visible details, making deviation mapping more challenging. The visual fidelity assessment methodology was therefore modified accordingly. For illustration purposes, we began by randomly selecting a ground truth UAV image and identified its corresponding camera position in Blender. The rendered image under this camera position was generated in Blender to match the pixel resolution of the ground truth UAV image (5472 × 3648 pixels). The focal length was manually tuned to be 24 mm to match the depth of the scene. Figure 18 shows the selected camera location in Blender (orange cone) and its associated rendered view of the roof (red roof area). The ground truth and rendered images used for the fidelity evaluation are shown in Figure 19a and c, respectively.

Next, a 200 × 200 pixels image patch was extracted from both ground truth (Figure 19a) and rendered (Figure 19c) images, focusing on a localized area with a water stain as shown in Figure 19b,d. Unlike the previous bridge case, where feature matching was performed across the entire image, this validation focused on registering small localized patches. To do so, feature detection and matching were applied between Figure 19b and Figure 19d, as shown in Figure 19e. Based on the initial matched features, we then computed a projective geometric transformation matrix. Any matched features that did not adhere to this transformation are considered outliers and were filtered out. All remaining inliers are shown in Figure 19f. Thereafter, the established transformation matrix was applied to the rendered patch (Figure 19d) to generate the recovered rendered patch, as shown in Figure 19g.

Because the geometric transformation was applied to a small image patch instead of the entire image view, visible gaps appeared along the top and left boundaries of the recovered rendered image in Figure 19g. To prevent these edge artifacts from impacting subsequent processing, we applied a filtering step. First, we calculated the absolute pixel-wise intensity difference between the ground truth (Figure 19b) and recovered rendered (Figure 19g) image patches, resulting in a grayscale difference map as shown in Figure 19h. Thereafter, we defined a ROI in Figure 19h to mask out areas outside this region by setting their pixel values to black (0 intensity), as shown in Figure 19i. Finally, the deviation map (Figure 19i) was converted into the RGB heatmap and was further overlaid with the ground truth patch (Figure 19b), leading to the final results shown in Figure 19j with a magnified view in Figure 19k.

**Figure 20:**
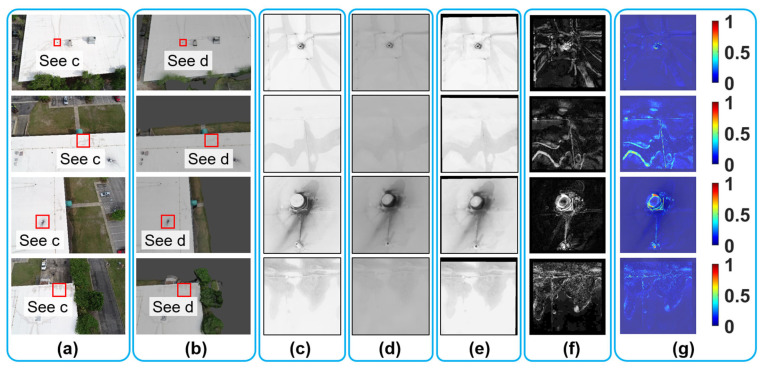
Evaluation results for four different camera positions: (**a**) ground truth UAV images; (**b**) corresponding rendered images; (**c**) grayscale ground truth image patches cropped from ground truth UAV images; (**d**) grayscale patches cropped from rendered images; (**e**) recovered rendered image patches after histogram matching and geometric transformation; (**f**) absolute pixel-wise comparison between (**c**) and (**e**); (**g**) final evaluation results with color plots superimposed on the ground truth patches.

Following this protocol, we selected 12 different roof locations for visual fidelity assessment. Due to the limited space, only the results of four locations are shown in this subsection, while the results for the remaining eight roof locations can be found in Appendix B. Figure 20 shows the results from the first four roof locations. Column a of the figure displays the ground truth UAV images, while column b shows the corresponding rendered images in Blender. Small image patches ranging from 200 × 200 pixels to 600 × 600 pixels were selected from both ground truth and rendered images, as shown in Figure 20c and d, respectively. These patches focused on water stains or other surface textures.

To prepare the image for comparison, we first applied histogram matching to align the brightness and contrast of the rendered patches (Figure 20d) with the ground truth patches (Figure 20c). Then, geometric transformations were applied to align the image coordinates. The results after these modifications yielded recovered rendered patches, which are shown in Figure 20e. Following this, we subtracted the absolute pixel-wise intensity between the ground truth patch (Figure 20c) and recovered rendered patch (Figure 20e), resulting in the deviation map shown in Figure 20f. Lastly, the visual fidelity assessment results can be found in Figure 20f, where the RGB heatmaps were overlaid with ground truth patches (Figure 20c).

**Figure A3:**
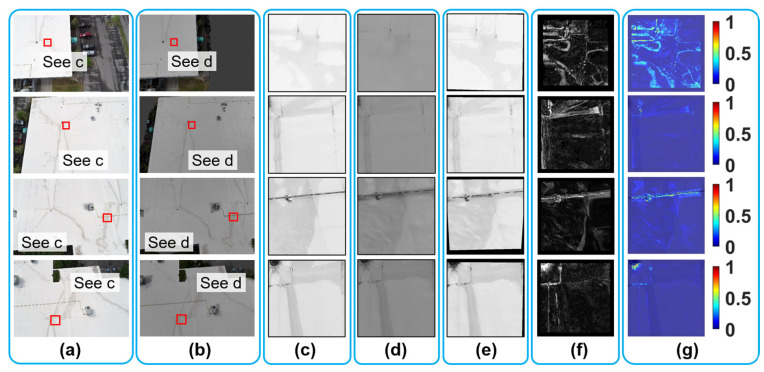
Evaluation results for four different camera positions: (**a**) ground truth UAV images; (**b**) corresponding rendered images; (**c**) grayscale ground truth image patches cropped from ground truth UAV images; (**d**) grayscale patches cropped from rendered images; (**e**) recovered rendered image patches after histogram matching and geometric transformation; (**f**) absolute pixel-wise comparison between (**c**) and (**e**); (**g**) final evaluation results with color plots superimposed on the ground truth patches.

**Figure A4:**
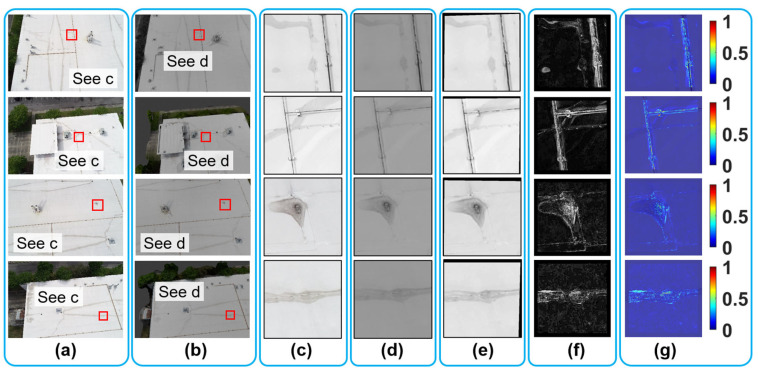
Evaluation results for four different camera positions: (**a**) ground truth UAV images; (**b**) corresponding rendered images; (**c**) grayscale ground truth image patches cropped from ground truth UAV images; (**d**) grayscale patches cropped from rendered images; (**e**) recovered rendered image patches after histogram matching and geometric transformation; (**f**) absolute pixel-wise comparison between (**c**) and (**e**); (**g**) final evaluation results with color plots superimposed on the ground truth patches.

In summary, the results of the four representative roof locations (Figure 20), along with the eight remaining locations included in Appendix B (Figure A3 and Figure A4) demonstrate that the VR model of the building exhibits medium to high visual fidelity across most roof areas. However, certain localized regions show lower fidelity, indicated by yellow to red coloring. For example, in the third row of Figure 20, a non-structural rooftop equipment unit is identified as an area of low fidelity. This discrepancy likely arises from limited UAV image coverage of that equipment unit during field data collection, resulting in reconstruction inaccuracies in the photogrammetric model of the building. Additional discussion on the sources of errors can be found in Section 8.1.

## 8. Discussion

### 8.1. Discussion of Two Validation Studies

In terms of VR model development, results from Section 6.1 and Section 7.1 demonstrate the effectiveness of this proposed framework in prototyping interactive VR environments for civil infrastructure. In contrast to most existing VR studies that rely on Unity [31] for VR interface development [12,13,14,16,17], our approach integrates Blender for lighting enhancement and 360-degree image rendering, and VIAR360 for immersive scene construction. VIAR360 provides a user-friendly, web-based interface that significantly reduces the need for custom software development. This combined use of Blender and VIAR360 represents a novel and accessible alternative for VR development for structural inspection, not yet reported in the existing literature.

In terms of VR model assessment, Section 6.2 and Section 7.2 report the effort to align and process the ground truth and Blender-rendered images using a combination of image registration, histogram matching, and pixel-level deviation mapping to identify fidelity discrepancies. The assessment results confirm that the VR models of the stone arch bridge and the steel campus building are overall reliable representations of their physical counterparts. This finding also suggests that the proposed VR development approach successfully preserves surface texture and visual detail critical for structural inspection. To the best of our knowledge, this is the first study to rigorously quantify the visual fidelity of VR models in the context of civil infrastructure inspection.

The deviation map used in this study provides a spatially intuitive visualization of visual fidelity, highlighting where and to what extent the rendered VR model deviates from the real-world UAV image. This spatial mapping allows engineers to identify localized areas of high or low fidelity that may affect damage inspection later on. Given this context, defining a single numerical threshold within the deviation map below which VR-based inspection becomes unreliable may be overly simplistic and potentially misleading. A threshold offers only a scalar value, whereas visual fidelity is inherently spatial and heterogeneous across a structure’s surface. For instance, small deviations near a critical crack may be more critical than larger deviations in unimportant background areas.

There are a few factors that could contribute to the visual discrepancies between the ground truth and Blender-rendered images. First, UAV images may inherently contain lens distortions and perspective errors. These distortions, if uncorrected, could potentially introduce minor geometric inconsistencies when compared with rendered images from VR models. Distortion-corrected UAV imagery using established camera calibration techniques [70] could improve the visual fidelity assessment results. Another contributing factor is the inherent smoothing and interpolation during SfM-MVS reconstruction [71], which can reduce the sharpness of fine surface features. While we did not apply any post-processing to enhance surface sharpness of the VR model in this study, edge-preserving sharpening algorithms [72] could potentially improve local feature clarity in the rendered VR models. Lastly, UAV image coverage also plays a role. For example, areas that were difficult to access or were not prioritized during flight planning had fewer overlapping images covered. This resulted in sparse photogrammetric data for those regions, leading to reconstruction artifacts and lower texture quality in the final VR models.

### 8.2. Limitations

This study has several limitations. First, photogrammetry presents challenges when reconstructing thin features, such as the green plants in the mid-span of the bridge façade (Figure 7a). This limitation, common to SfM-MVS [26], could be overcome by incorporating higher-precision 3D scanning technologies such as LiDAR. Second, the fidelity evaluation in this study relied on comparisons between rendered images from VR models and UAV images. First-person screenshots from a VR headset were not included due to the difficulty of aligning headset views with UAV camera perspectives. Future studies should explore methods to capture and align in-headset views for evaluation. Third, the sensitivity and robustness of our fidelity assessment method against varying lighting conditions, image registration errors, and different texture resolution of the VR models remain to be fully validated. Our current evaluation is based on real-world field data, where true pixel-level ground truth is inherently unavailable. Adopting controlled synthetic environments using real-world objects with known geometry and textures would allow us to simulate UAV image capture and VR rendering under varied lighting, texture, and resolution conditions. Such a setup would enable quantitative benchmarking of the fidelity metric and deeper insight into how different types of reconstruction or rendering artifacts affect the deviation maps. Fourth, the validations were limited to two testbeds: a historic stone arch bridge and a single-story steel building. While diverse in structure types and materials, these examples do not cover the full range of civil infrastructure. For example, our method may fail in reconstructing structures with highly obstructed surfaces or minimal visual detail. Lastly, the implementation of the proposed fidelity assessment approach in practical field operations may depend on costs, required expertise, and equipment availability.

### 8.3. Future Work

While this study presents a pipeline for VR model development and visual fidelity assessment, future efforts could focus on automating several stages of the process such as the workflows in Blender and VIAR360. In addition, the methodology in this study could be extended to the integration of artificial intelligence (AI) with VR to assist in automatic damage detection, classification, or prioritization. For example, machine learning models [73] could identify crack patterns or surface degradation and embed annotations directly into the VR environment. Additionally, manual annotations by inspectors, such as marking damage locations or tagging concerns during a VR walkthrough, could enhance collaboration and recordkeeping. Recent efforts in cultural heritage inspection have demonstrated the feasibility of AI-assisted visual inspection in immersive settings [74,75], suggesting promising synergies between VR and AI for structural assessment.

Future work should also expand toward a more human-centered and interdisciplinary assessment in VR-based structural inspection. While this study focused on the visual fidelity evaluation of VR models using computational, algorithm-driven frameworks, the psychological and experiential impacts of VR for end users (e.g., inspectors) remain largely unexplored. In related domains involving inspection-like tasks outside the civil engineering field, VR has been reported to offer several psychological benefits such as creating immersive 3D environments [76,77,78], reducing psychological stress [79,80,81], and enhancing model visualization [82,83,84]. Nevertheless, these findings have not been systematically investigated in the context of civil infrastructure inspection. Future research should therefore conduct controlled experiments comparing VR-based and traditional inspection workflows to evaluate user performance, decision-making accuracy, and cognitive load.

## 9. Conclusions

In this study, we present a comprehensive framework to address a critical challenge in VR-based structural inspection: can VR models accurately reflect their real-world counterparts? We started the investigation by first reviewing the related work, identifying the research gap, and defining the scope of the study. Thereafter, we proposed a methodology for developing an accessible VR production workflow. Next, we introduced a novel pixel-level visual fidelity assessment approach to evaluate the established VR models. Both VR development and assessment methodologies were validated through case studies of a stone arch bridge and a steel warehouse building. Results indicate that our work provides an interpretable, spatially explicit evaluation of the texture accuracy of VR models.

This study equips engineers and researchers with tools to systematically assess the visual quality of VR inspection environments before they are deployed for professional use. As immersive technologies become more prevalent in structural inspection and asset management, the ability to validate the accuracy of digital replicas will play a pivotal role in ensuring the reliability of virtual observations and the credibility of inspection outcomes. This work lays the groundwork for the broader adoption of fidelity-aware VR inspection systems and may inform future standards in digital twin verification, training simulations, and remote condition assessments across diverse civil infrastructure types.

## Figures and Tables

**Table 1:** Relevant literature work in VR-based structural inspection.

Authors	Journal/Proceeding	Publisher	Year	Reference
Napolitano et al.	MDPI Sensors	MDPI	2018	[15]
Attard et al.	IEEE IST Conference	IEEE	2018	[16]
Omer et al.	Structure and Infrastructure Engineering	Taylor & Francis	2019	[12]
Bacco et al.	IEEE Access	IEEE	2020	[17]
Omer et al.	Journal of Bridge Engineering	ASCE	2021	[13]
Luleci et al.	Automation in Construction	Elsevier	2024	[14]
Yiğit and Uysal	Measurement	Elsevier	2025	[18]

**Table 2:** Integration of scenes, hotspots, and interactive content into the VR user interface.

VR Scene	Camera Location	Theme	Common Features	Transition Hotspots
Scene 0	L0	Bridge overview; link to other scenes	Pre-defined point of view; background ambient sound	Visit Scenes 1, 2, 3, and 4
Scene 1	L1	Detailed inspections of a localized area at a close distance		Visit Scenes 0 and 2
Scene 2	L2			Visit Scenes 0, 1, and 3
Scene 3	L3			Visit Scenes 0, 2, and 4
Scene 4	L4			Visit Scenes 0 and 3

**Table 3:** Key terminologies in visual fidelity evaluation.

Terminology	Definition	Source
Ground truth	UAV-captured image taken directly from the field during site inspection.	UAV camera
Rendered	Image generated in Blender from the VR model under a matched camera position.	VR model
Recovered rendered	Rendered image after geometric transformation and histogram matching to align with the ground truth image.	Transformed in MATLAB [56]
Deviation map	Color-coded map showing per-pixel intensity difference between recovered rendered and ground truth images.	Extracted from registered images

## Data Availability

Some or all data, models, or code that support the findings of this study are available from the corresponding author upon reasonable request.

## Appendix A

Figure A1 and Figure A2 show the visual fidelity assessment results of the bridge VR model for the remaining eight camera positions. Ground truth and rendered images rendered in Blender are illustrated in columns a and b in both figures.

## Appendix B

Figure A3 and Figure A4 show the visual fidelity assessment results of the building VR model for the remaining eight camera positions. Ground truth and rendered images rendered in Blender are illustrated in columns a and b in both figures.
